# Comparative Studies on Ultraviolet-Light-Derived Photoresponse Properties of ZnO, AZO, and GZO Transparent Semiconductor Thin Films

**DOI:** 10.3390/ma10121379

**Published:** 2017-12-01

**Authors:** Chien-Yie Tsay, Wei-Tse Hsu

**Affiliations:** Department of Materials Science and Engineering, Feng Chia University, Taichung 40724, Taiwan; vm6jo4yk6@gmail.com

**Keywords:** oxide semiconductor, zinc oxide, sol-gel spin coating, MSM UV photodetector, photoresponse properties

## Abstract

ZnO, Al-doped ZnO (AZO), and Ga-doped ZnO (GZO) semiconductor thin films were deposited on glass substrates via a sol-gel spin-coating process for application in a photoconductive ultraviolet (UV) detector. The doping concentrations of Al and Ga were 1.0 at % in the precursor solutions. In this study, the microstructural features and the optical and electrical properties of sol-gel-derived ZnO, AZO, and GZO thin films were compared, and the performance of ZnO-based UV photodetectors under ultraviolet A (UVA) light were measured. Experimental results confirmed the synthesis of single-phase nanocrystalline ZnO-based thin films and the successful substitution of Al and Ga into Zn sites in ZnO crystals. The results also demonstrated that the optical transmittance and electrical properties of ZnO thin films could be improved by Al and Ga doping. UV photodetectors based on ZnO-based thin films, having a metal-semiconductor-metal (MSM) configuration, were fabricated with Al inter-digitated electrodes. All photodetectors showed an ohmic nature between semiconductor and electrode contacts and exhibited a sharp increase in photocurrent under illumination with UVA light. We found that the MSM UV photodetector based on the GZO semiconductor thin film exhibited the best UV response (*I*_UVA_/*I*_dark_) of 73.3 and the highest photocurrent responsivity of 46.2 A/W under UVA light (power density ~0.825 mW/cm^2^) at 5 V bias.

## 1. Introduction

The photodetector is the key optoelectronic component in smart life technologies and in many sensing systems that require detecting ultraviolet (UV) or visible light for turning on or off systems. Functional metal oxide semiconductors with a wide bandgap (>3 eV) have drawn much attention for the development of visible-blind, UV-sensitive photodetectors [1,2]. Semiconductor UV photodetectors operating only at a mild bias (low power requirement) and insensitive to magnetic fields have been widely utilized in optical communications, environmental monitoring, biological agents, and aerospace and military applications [3,4]. In recent years, metal-semiconductor-metal (MSM) structured UV photodetectors have attracted research interest due to their high photoconductive gain and photoresponsivity (amplifying equilibrium is not necessary), as well as their fabrication simplicity and suitability for monolithic integration [1,5].

Zinc oxide (ZnO) is an important n-type II-VI group semiconductor with a wide direct bandgap of 3.37 eV, as well as a high exciton binding energy of 60 meV at room temperature (RT). It is also thermally and chemically stable and has high radiation hardness, which extend its use in harsh environments [6,7]. Because of its wide bandgap and high exciton energy, ZnO is a suitable semiconductor material for the sensing layer in photodetectors, providing great sensitivity for UV radiation without extra filters, and ensuring effective excitonic emission at RT [1,3]. It is well known that n-type doped ZnO thin films are achieved by the substitution of Zn^2+^ cations with group III elements (B, Al, Ga, In) or group IV elements (Si, Ti, Sn) to improve the optical and electrical properties and enhance the stability of ZnO thin films by increasing their transparency in the visible region as well as their electrical conductivity [8]. The efficiency of the impurity doping is related to its electronegativity and differences between the ionic radii of the dopant element and Zn [9]. Both Al and Ga are the most suitable dopants for substituting into ZnO thin films in transparent electronics applications [10]. A few papers have compared the physical properties of Al-doped ZnO (AZO) and Ga-doped ZnO (GZO) thin films prepared under the same processes and conditions [9,10,11,12,13]. Ng et al. reported that sol-gel-derived AZO with an Al doping level of 1 at % and a GZO with a Ga doping level of 2 at % were found to have the optimal electrical properties [11].

Several studies on the photosensing performance of solution-processed ZnO-based MSM photodetectors have demonstrated that the ability of photocurrent generation depends on the wavelength of the illumination light, and that the highest photosensitivity can be achieved under UVA light (wavelength range of 340 to 400 nm) illumination [4,14,15,16]. For example, Shaikh et al. reported that a fabricated ZnO-based MSM UV photodetector showed excellent responsivity of 185 A/W, with fast response and recovery times under UV illumination (wavelength ~365 nm) at 5 V bias voltage [15]. They also presented that ZnO-based photodetectors showed an evident response in the UV region of the spectrum and less response in the visible region. Inamdar et al. fabricated a photoconductive MSM UV photodetector based on spray-deposited ZnO thin film that showed a relatively high photocurrent (1.3 mA) and fast switching. They also reported that the maximum response was found in the wavelength range of 350 to 370 nm, with a sharp cut-off wavelength at 375 nm [4]. Shinde et al. prepared UV photoconductive detectors using a Ga-doped ZnO sensing layer grown by spray pyrolysis and achieved the highest responsivity at a wavelength of 365 nm and 5 V bias [16].

Many processing techniques, such as magnetron sputtering, pulsed laser deposition, chemical vapor deposition, and solution processes, have been used for the fabrication of high-quality impurity-doped ZnO thin films [17]. The sol-gel route is a good choice for cost-efficiency, larger-area deposition, and good composition controllability of device-quality impurity-doped ZnO thin films [16]. In this study, ZnO, AZO, and GZO transparent semiconductor thin films were fabricated by a sol-gel spin-coating process on glass substrates and incorporated into MSM UV photodetectors. The goals of this study were to investigate and compare the influences of Al or Ga single doping on the physical properties of ZnO thin films and the photosensing characteristics of three kinds of ZnO-based photodetectors under UVA illumination.

## 2. Materials and Methods

### 2.1. Thin Film Preparation and UV Photodetector Fabrication

**Figure 1:**
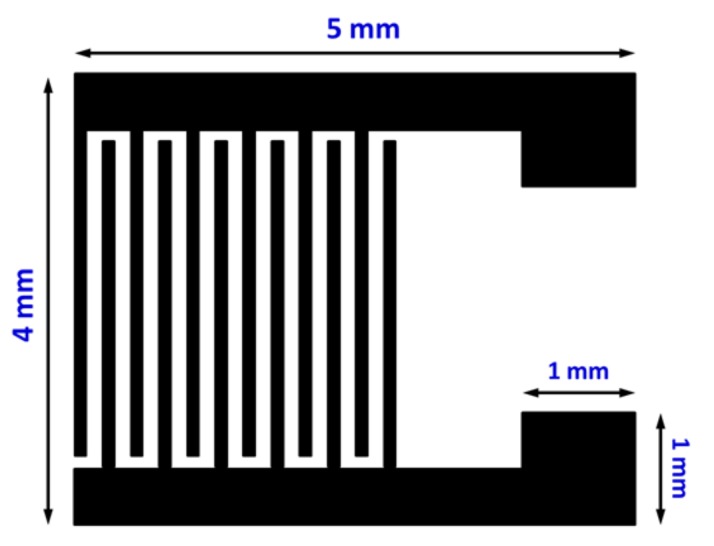
Schematic pattern of interdigital (IDT) electrodes of the ZnO-based metal-semiconductor-metal (MSM) photodetector.

The ZnO, Al-doped ZnO (AZO), and Ga-doped ZnO (GZO) thin films employed as the sensing layer of the UV photodetectors were grown on alkali-free glasses (NEG OA-10) by the sol-gel method using the spin-coating technique. Analytical reagent (AR) grade zinc acetate, aluminum nitrate, and gallium nitrate were used as a source for Zn, Al, and Ga, respectively. The concentration of metal ions in the resultant solution was 0.5 M, and the molar ratio of metal ions to diethanolamine (DEA) was 1.0. The dopant concentration of doping samples, defined as [M]/[Zn+M] atomic ratio, where M is Al or Ga, was maintained at 1 at % in AZO and GZO precursor solutions. Each precursor solution was synthesized by mixing a stoichiometric amount of inorganic metal salts in 2-methoxyethanol (2-ME), after which DEA was added into that solution as a stabilizer. Each mixture was stirred with a magnetic stirrer at 60 °C for 2 h and held for 72 h of aging before being used as the coating solution. The spin coating was carried out at 1000 rpm for 30 s, followed by heating at 300 °C for 10 min to evaporate the solvent and remove organics before the next layer was applied. This procedure was performed three times, and the resulting dried sol-gel films were annealed at 500 °C for 1 h to form the crystalline oxide films. For the fabrication of the metal-oxide-metal (MSM) photodetector after the growth of the ZnO-based thin film on a glass substrate, Al inter-digitated electrodes (150 nm thick) were patterned onto the thin film with a shadow mask by vacuum thermal evaporation. The working pressure of the thermal evaporation process was controlled at about 1 × 10^−6^ Torr, and the substrate temperature was kept under 90 °C during deposition. The electrode pattern of the photodetector consisted of two inter-digitated electrodes with a finger width of 100 μm and spacing between the fingers of 150 μm; the configuration is illustrated in Figure 1.

### 2.2. Property Characterization

The crystal structures of the as-prepared ZnO-based thin films were identified by X-ray diffraction (XRD) using an X-ray diffractometer (D8 SSS, Bruker, Karlsruhe, Germany) with CuKα radiation. The microstructural features of the films were investigated using a scanning electron microscope (SEM, S-4800, Hitachi High-Technology, Tokyo, Japan); the topography and surface roughness of the films were examined by a tapping mode scanning probe microscope (SPM, NS4/D3100CL/MultiMode, Digital Instruments, Mannheim, Germany). The transmission and reflection spectra measurements of the glass/thin film samples were carried out using an ultraviolet-visible (UV-Vis) spectrophotometer (U-2900, Hitachi High-Technology, Tokyo, Japan). The electrical properties of the films were measured by a Hall measurement system (HMS-3000, Ecopia, Gyeonggi-do, Korea) using the van der Pauw configuration. The UV sensing and time-dependent photoresponse measurements of the photodetectors were measured at RT in ambient conditions using a Source-Measure Unit (Jiehan 5000, JIEHAN Technology Corporation, Taichung, Taiwan) with illumination provide by a UVA mercury lamp (wavelengths of 315–400 nm) with a light density of 0.825 mW/cm^2^.

## 3. Results and Discussion

### 3.1. Structural Features, Optical and Electrical Properties of Oxide Thin Films

**Figure 2:**
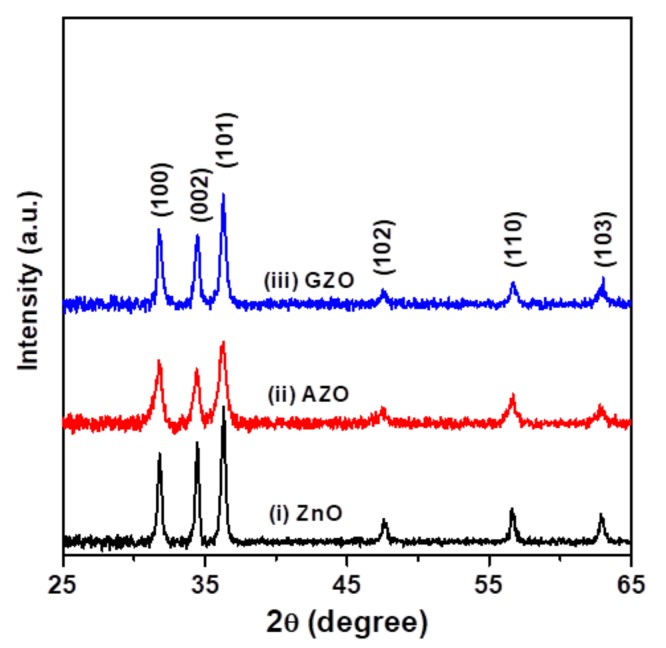
X-ray diffraction (XRD) patterns of (**i**) ZnO, (**ii**) AZO, and (**iii**) GZO thin films deposited on glass substrates by a sol-gel spin-coating process.

The XRD patterns of as-prepared ZnO, AZO, and GZO thin films on glass substrates (Figure 2) revealed that they were polycrystalline thin films. The diffraction peaks well matched Joint Committee on Powder Diffraction Standards (JCPDS) card No. 036-1451, indicating that the three ZnO-based thin films had a hexagonal wurtzite structure with a *P63mc* space group. XRD studies also revealed that the Al and Ga ions were successfully incorporated into the ZnO lattice, without the formation of a second phase. In addition, the full widths at half-maximum (FWHMs) for the three major diffraction peaks, (100), (002), and (101), of the two impurity-doped thin films (patterns (ii) and (iii) in Figure 2) were wider than those of the undoped thin film (pattern (i) in Figure 2), implying that the front had a finer crystallite size. For example, the FWHMs of (101) peaks for ZnO, AZO, and GZO were 0.34°, 0.60°, and 0.38°, respectively. The peak broadening of nanocrystalline ZnO-based materials is related to lattice strain and crystallite size [18]. Jun et al. reported that the compressive stress in the direction of the *c*-axis of sol-gel-derived AZO and GZO thin films increased with increasing dopant concentration (0.5 to 2.0 mol %) and that there was little change in GZO thin films [13]. Results of calculated stress also showed the 1.0 mol % Al- or Ga-doped ZnO thin films had a close compressive stress value approaching −0.9 GPa. In addition, it is well known that the crystallite size of polycrystalline materials can be estimated from 2θ and the FWHMs of the diffraction peaks using Scherrer’s formula. The average crystallite sizes of ZnO, AZO, and GZO, for the three major diffraction peaks of (100), (002), and (101), were 26.1 nm, 16.2 nm, and 22.5 nm (Table 1). It was found that incorporating dopants into the ZnO crystal reduced the average crystallite size, and the AZO sample exhibited the finest average crystallite size.

The crystallite size of impurity-doped ZnO thin films varies because of differences in the ionic radii of the dopants and Zn; tetrahedral coordination leads to changes in the distance between both Zn-O and M-O (M: doping element) [7,19]. The incorporation of the impurities into ZnO crystals not only caused lattice distortion and non-uniform stress distribution, but also tended to create dopant-induced nucleation centers and lattice defects [10]. The difference between the ionic radius of Ga^3^^+^ (0.62 Å) and that of Zn^2^^+^ (0.74 Å) is smaller than the difference between the ionic radius of Al^+3^ (0.54 Å) and that of Zn^2^^+^ (0.74 Å) [13]. Thus, Ga^3^^+^ can easily be substituted for Zn^2^^+^ in the ZnO crystal with less lattice distortion than that caused by Al^3^^+^. Moreover, it is possible that Al ions may occupy both substitutional and interstitial sites in wurtzite crystals, whereas Ga ions occupy only substitutional sites [10].

**Figure 3:**
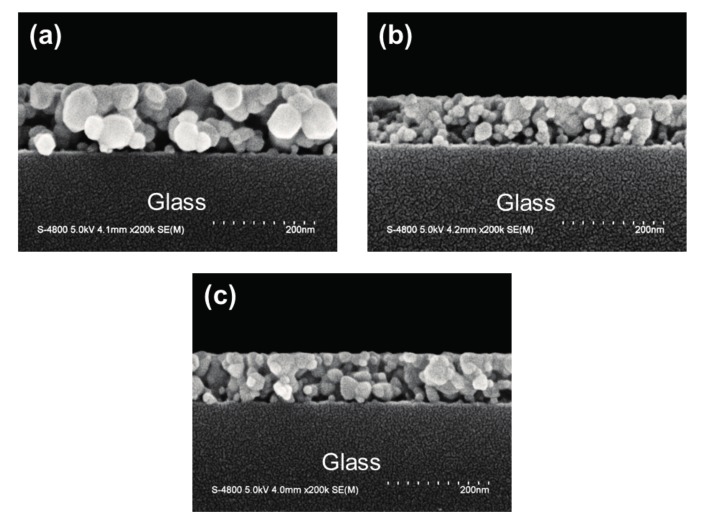
Cross-sectional scanning electron microscope (SEM) micrographs of ZnO-based thin films. (**a**) ZnO; (**b**) AZO; and (**c**) GZO thin films.

Figure 3 shows cross-sectional view SEM micrographs of the three ZnO-based thin films on glass substrates. These SEM images reveal that the polycrystalline ZnO-based thin films had an observably granular structure and uniform thickness, and that the particle sizes of the thin films were significantly reduced by introducing impurities (dopants). The mean film thicknesses of the ZnO, AZO, and GZO thin films were evaluated from corresponding SEM images to be 135 nm, 95 nm, and 98 nm, respectively, depending on the viscosity of the coating solution and spin-coating parameters. Srinatha et al. explained that the thickness of impurity-doped ZnO thin films decreases because of the decrease in the particle size [7]. Each SEM image also shows pores inside the sol-gel-derived ZnO-based thin films, the formation of which may be ascribed to the decomposition of residual organics in dried sol-gel films [20]. The porosity is one of the major reasons why the properties of solution-processed oxide thin films are inferior to those of sputtered oxide thin films. However, it may make sol-gel-derived ZnO-based thin films serviceable for applications in gas sensors and photodetectors.

**Figure 4:**
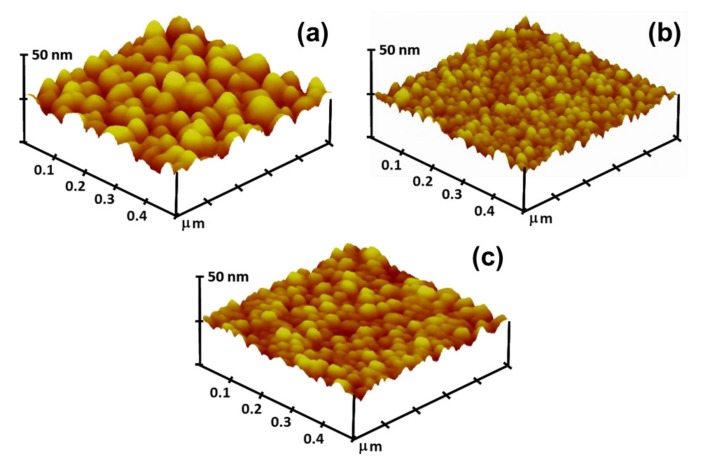
Surface scanning probe microscope (SPM) images of ZnO-based thin films. (**a**) ZnO; (**b**) AZO; and (**c**) GZO thin films.

The effects of Al and Ga doping on the surface morphologies of the films are presented by SPM images with a scan area of 0.5 μm × 0.5 μm (Figure 4). Each image shows significant particle configuration, and the dense and crack-free surfaces consisted of close-packed particles. The particles in the undoped thin film were observably larger than those in the impurity doped thin films, and the AZO thin film exhibited the finest particles. The average particle sizes of the ZnO, AZO, and GZO thin films, as determined from SPM images, were 35.9 nm, 23.8 nm, and 29.4 nm, respectively. The observation of the structural feature by SPM was in good agreement with the XRD and SEM results. Table 1 summarizes the measured values of surface root mean square (RMS) roughness of the three kinds of ZnO-based thin films. As can be determined from the table, Al and Ga doping reduced the RMS roughness, and the AZO thin film exhibited the lowest RMS value of 2.86 nm. The surface conditions of polycrystalline oxide thin films are strongly dependent on their microstructural features, especially the particle size. The above discussions of the XRD and SEM results support that explanation.

**Figure 5:**
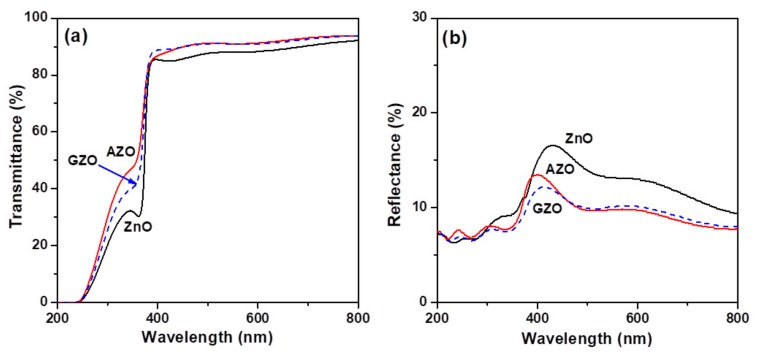
(**a**) Optical transmittance spectra and (**b**) optical reflection spectra of ZnO, AZO, and GZO thin films.

The optical transmission and reflection spectra of the three glass/oxide thin film samples in the 200–800 nm region are shown in Figure 5a,b, respectively. The thin film samples exhibited high transparency (*T* > 88.5%) in the visible region and had a significant absorption edge in the UV region. Recorded optical spectra revealed that the two impurity-doped thin film samples exhibited higher optical transmittance and lower reflectance over a range of wavelengths, 400–800 nm, as compared to the undoped thin film sample. The increase in transmittance and decrease in scattering resulted from the reduction in surface roughness. The calculated values of average transmittance and reflectance are listed in Table 1. The AZO thin film had the highest average transmittance of 91.6% and the lowest average reflectance of 9.55%. The improvement in the transparency of the impurity-doped thin films is attributed both to the smoothness of the surface and the reduction in thickness of the films.

**Figure 6:**
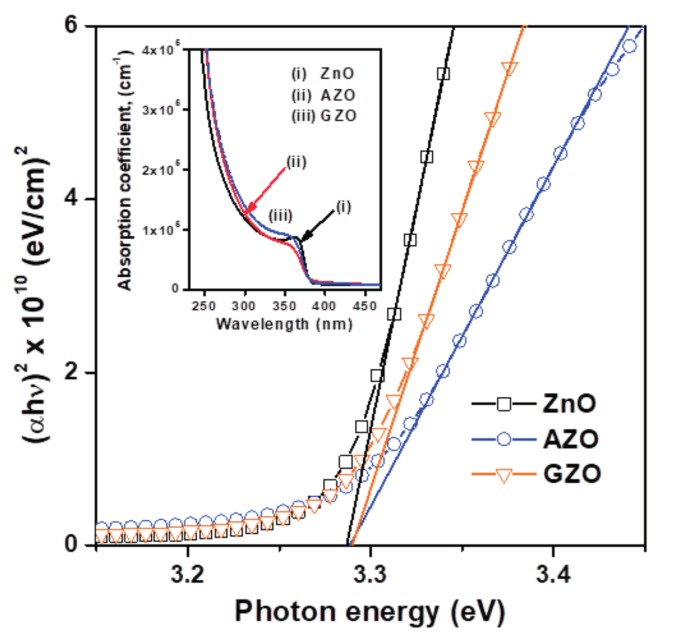
Plot of (*αhν*)^2^ versus photon energy (*hν*) of ZnO, AZO, and GZO thin films. In the inset, the plot of absorption coefficient with wavelength close to absorption edge is presented.

A study of optical transmission and reflection also allowed us to determine the absorption coefficient (*α*) and optical bandgap energy (*E*_g_). The measured transmittance (*T*) and reflectance (*R*) data were converted into the absorption coefficient (*α*), using the relationship *α*(*λ*) = 1/*d* ln[(1 − *R*^2^)/*T*], where *d* represents film thickness [21,22]. Variation of the absorption coefficient with the optical wavelength of the films is shown in the inset of Figure 6. In addition, the absorption coefficient (*α*) as a function of photon energy (*hν*) can be expressed as (*αhν*)^2^ = *A* (*hν* − *E*_g_) for a direct transition, where *A* is an energy-independent constant. The dependences (*αhν*)^2^ versus photon energy (*hν*) for the three ZnO-based thin films are plotted in Figure 6. The optical bandgap energy (*E*_g_) was determined by extrapolating the strain-line section of the curve from the plot of (*αhν*)^2^ versus photon energy (*hν*) to intercept the photon energy axis at (*αhν*)^2^ equal to 0. The estimated optical bandgap energies are presented in Figure 6; they were about 3.29 eV. This result is close to that of the sprayed ZnO thin films (3.285 eV) reported by Salah et al. [19]. The optical bandgap energy of the as-prepared ZnO-based thin films (~3.29 eV) was slightly red shifted as compared with the bulk ZnO (3.37 eV). This variance can be ascribed to the sol-gel-derived thin film samples consisting of nanosized crystals and the micro-strain induced in such films [7].

The results of the Hall effect measurement for comparison of the electrical properties among ZnO, AZO, and GZO thin films are listed in Table 1. The mean electron concentration and mean Hall mobility of the two impurity-doped samples were higher than those of the undoped sample, and the GZO sample exhibited the best electrical properties among the thin film samples. The increases in electron concentration of the AZO and GZO thin films were attributed to the substitution of Al^3^^+^ and Ga^3^^+^ ions at Zn^2^^+^ cation sites [8,13]. The effective substitution of the dopant (group III element) ions in Zn ions at their lattice positions can be described by the following equation [23]:

(1) $$M_{2}O_{3(S)}=2M_{Zn•}+2O_{O}+1/2O_{2(g)}+2e^{\prime }$$

where *M_Zn_*_•_ is the dopant substituted for Zn^2^^+^ in the ZnO matrix and *O_O_* is the oxygen from the lattice [24]. The electron concentration and Hall mobility of the AZO thin films were lower than those of the GZO thin films because the AZO thin films had a finer grain size and larger grain boundary area. In addition, the resistivity (*ρ*) and electron concentration (*n*) of an n-type semiconductor have an inverse relationship (*ρ* ∝ 1/*n*), so the high electron concentration density of the thin films led to the low resistivity.

### 3.2. UV Sensing and Photoswitching Studies of Photodetectors

**Figure 7:**
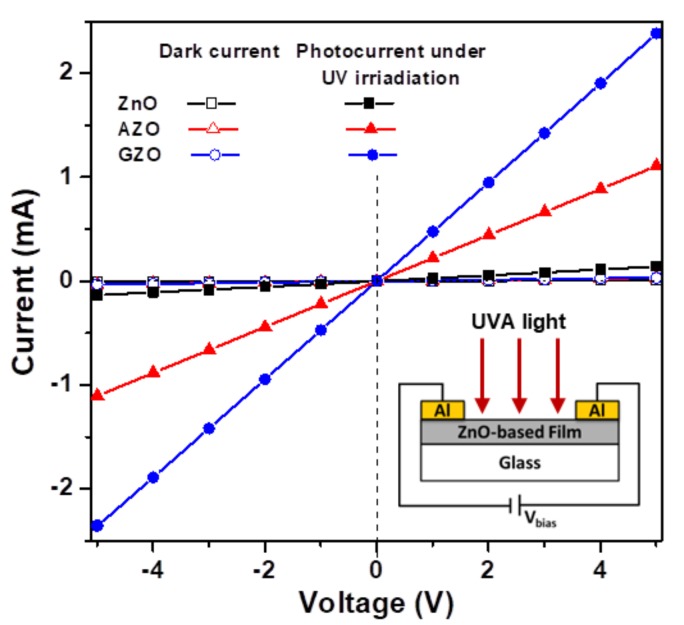
Current-voltage (*I*-*V*) characteristics of dark and photoilluminated currents under irradiation with UVA light obtained from ZnO-based MSM photodetectors. The inset in the bottom right corner shows the arrangement for *I*-*V* measurements.

Figure 7 presents the current-voltage (*I*-*V*) characteristics of fabricated ZnO-based MSM photodetectors with Al as contact electrodes both in the dark and under exposure to UVA light. The inset of Figure 7 presents the structure of the MSM (Al-ZnO-Al) photodetector. All devices exhibited linear *I*-*V* characteristics under both forward and reverse bias, which indicated the ohmic nature of the contact of the Al electrode on the ZnO-based semiconductor thin film and showed that they were photoconductive UV detectors. Yu et al. proposed that high-density interface states will form at the ZnO/electrode interfaces during the deposition of the metal electrodes and may facilitate electron tunneling through the barrier between metal electrode and oxide semiconductor. Thus, the ohmic contact will form at the semiconductor/electrode junction [14].

The measured dark currents (*I*_dark_) were 9.2 μA, 31.3 μA, and 32.6 μA for the ZnO, AZO, and GZO samples, respectively, at 5 V bias. The two impurity-doped samples exhibited dark currents higher than that of the undoped sample because the incorporation of Al or Ga dopants at Zn lattice sites in ZnO creates donor levels in the forbidden gap, thereby increasing the electron concentration density and the level of dark current. Under illumination by UVA light with an average intensity of 0.825 mW/cm^2^, the electron hole pairs were created by the absorption of UVA radiation, and the photocurrent (*I*_UVA_) of corresponding samples jumped to 0.14 mA, 1.11 mA, and 2.38 mA (Figure 7). The sample had a high photocurrent and was expected to have low contact resistance [15]. The ability of photoelectrical conversion can be evaluated with the ratio of photocurrent-to-dark current (*I*_UVA_/*I*_dark_) [25]. The calculated results of *I*_UVA_/*I*_dark_ (Table 2) revealed that the GZO device had high UVA-sensitive photoconductivity and a strong photoelectrical conversion ability.

**Figure 8:**
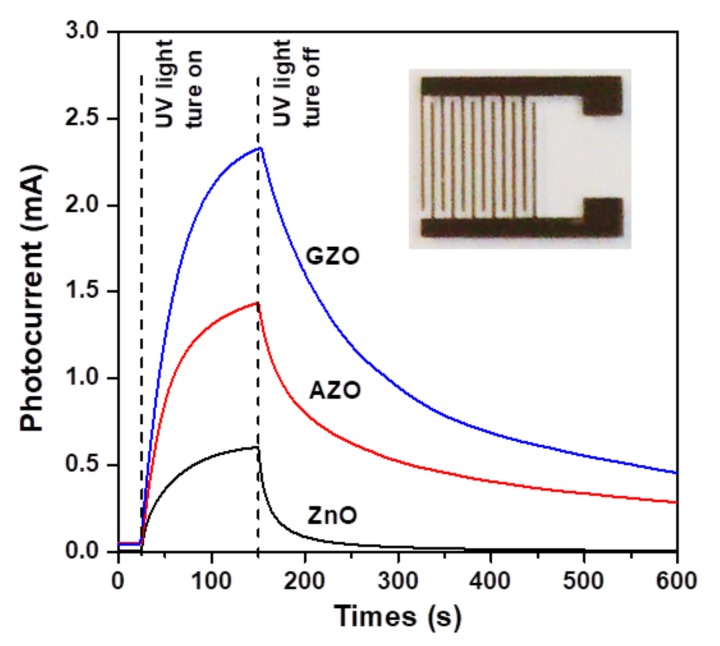
Photoresponse of the ZnO-based MSM photodetectors at a bias of 5 V under illumination with UVA light. The inset shows a top view optical microscope (OM) image of the device.

The time-dependent photoresponse characteristics of the three ZnO-based photodetectors exposed under UVA light and fixed at 5 V bias are presented in Figure 8. The measured current before the UVA light was turned on is indicated as *I*_off_ (off current). When the UVA light was turned on, the photocurrents of all the photodetectors were found to increase rapidly with exposure time and thereafter to achieve a specific saturation level, indicated as *I*_on_ (on current). When the UVA illumination was turned off, the photocurrent significantly decreased and tended to return to the initial state. The photoresponse behavior (Figure 8) of the sol-gel-derived ZnO-based MSM UV photodetectors is consistent with previous reports and is an exponential function [4,26]. In addition, it is noted that the photocurrent did not reach its initial value due to the persistent photoconductivity effect (PPC) [27]. The PPC effect the photocurrent of the detectors continued to flow even after the UV light source was turned off [15].

The photoresponse of the wide-bandgap oxide semiconductors to UV light illumination was a combination of two phenomena [5,14]. After exposure to UV light, electron-hole pairs were generated by the absorption of photons and increased the carrier concentration of the oxide semiconductors. In addition, the photogenerated holes recombined with the trapped electrons at oxygen defect sites, and the photogenerated electrons increased the electron concentration in the conduction band. These two phenomena thereby caused an increase in the value of the photocurrent. When the UV light was turned off (recovery period), oxygen molecules were adsorbed on the surface layer of the oxide semiconductors due to capture by free electrons, creating a depletion region near the surface that could reduce the conductivity and decrease the photocurrent back to its initial value [19]. It is noted that the AZO and GZO devices exhibited a slow decay behavior during the recovery period. Liu et al. reported that the ZnO photoconductive detectors exhibited a slow decay time, perhaps mainly due to the excess lift time of trapped holes [28].

The main parameters for describing or evaluating the performance of UV photodetectors include photoconductivity gain (*G*), sensitivity (*S*), and photocurrent responsivity (*PR*) [15,19,26]. The calculated results for the three kinds of photodetectors are summarized in Table 2. According to the photoswitching characteristic of the devices, the photoconductivity gain (*G*) is defined as:

(2) $$G=(I_{on}/I_{off})$$

where *I_on_* and *I_off_* are the recorded values of on current and off current of the photodetector. The calculated results of the photoconductivity gains (*G*) of the ZnO, AZO, and GZO photodetectors were 67.0, 28.7, and 51.8, respectively. Although the two impurity-doped samples exhibited higher photocurrents than the undoped sample, the increase in the off currents led to a decrease in photoconductivity gain [24]. Sensitivity (*S*) (defined as the photocurrent (*I_ph_*) per off current) is expressed as:

(3) $$S=(I_{ph}/I_{off})$$

where *I_ph_* = *I_on_* − *I_off_*. The influence of the chemical composition of the sensing layer on the sensitivity showed the same tendency as photoconductivity gain. In addition, the photocurrent responsivity (*PR*) (defined as the photocurrent (*I_ph_*) per incident optical power) is expressed as:

(4) $$PR=(I_{ph}/P_{opt})$$

where *P_opt_* is the power density of the illumination light. The photocurrent responsivities of the ZnO, AZO, and GZO devices were calculated to be 12.0, 28.0, and 46.2 A/W. Such results clearly show that the GZO photodetector had the highest responsivity (46.2 A/W), as compared to the performance of a ZnO UV photoconductive detector (30.0 A/W, at 3 V bias) fabricated on quartz by radio frequency magnetron sputtering [28]. The high responsivity of a photodetector can be attributed to its high photoconductivity.

## 4. Conclusions

Transparent semiconductor thin films of ZnO, AZO, and GZO were prepared by a sol-gel spin-coating process, and ZnO-based MSM UV photodetectors were fabricated. Results of UV-Vis and Hall effect measurements revealed that Al and Ga doping can both significantly improve the optical and electrical properties of sol-gel-derived ZnO thin films. The average transmittance of the two impurity-doped ZnO thin films was >91%, and the average reflectance was <10%. The GZO transparent semiconductor thin films had the lowest mean resistivity of 1.22 × 10^2^ Ω cm and the highest mean Hall mobility of 11.0 cm^2^/Vs, so Ga doping seems to be more effective than Al doping. The *I*-*V* characteristics of the photodetectors showed that the three ZnO-based MSM UV photodetectors operated in the photoconductive mode and indicated that the GZO photodetector had a strong ability of photoelectrical conversion. Under illumination with UVA light, the GZO photodetector had the best UV response of 73.3 and the highest photocurrent responsivity of 46.2 A/W at 5 V bias.

## Figures and Tables

**Table 1:** Structural, optical, and electrical properties of sol-gel-derived ZnO, AZO, and GZO thin films.

Thin Film Sample	Average Crystallite Size (nm)	Root Mean Square (RMS) Roughness (nm)	Average Transmittance (%) ^a^	Average Reflectance (%) ^b^	Mean Electron Concentration (cm^−3^)	Mean Hall Mobility (cm^2^/Vs)	Mean Resistivity (Ω-cm)
ZnO	26.1	5.13	88.6	12.8	1.84 × 10^14^	4.76	7.71 × 10^3^
AZO	16.2	2.86	91.6	9.55	2.52 × 10^15^	6.09	4.14 × 10^2^
GZO	22.5	3.08	91.4	9.75	5.09 × 10^15^	11.0	1.22 × 10^2^

The average transmittance values ^a^ and average reflectance values ^b^ were calculated from the transmittance and reflectance data of wavelengths from 400 to 800 nm.

**Table 2:** Summary of the photosensing and photoswitching characteristics, and photoresponsivity of ZnO-based MSM photodetectors.

Sensing Layer	UV Response (*I*_UVA_/*I*_dark_)	Photoconductivity Gain (*I*_on_/*I*_off_)	Sensitivity (%)	Photocutrrent Responsivity (A/W)
ZnO	15.3	67.0	66.0	12.0
AZO	35.5	28.7	27.7	28.0
GZO	73.3	51.8	50.8	46.2
